# Bridging the academic–clinical gap: a comparative cross-sectional analysis of self-reported evidence-based practice engagement and associated research activity between fourth-year nursing students and interns

**DOI:** 10.3389/fmed.2026.1862936

**Published:** 2026-07-29

**Authors:** Fathia Ahmed Mersal, Manal Hassan Baqeas, Abdulhamid Gharib Alrwili, Boshra Attia Mohammed, Ohoud Naif Aldughmi, Enas Mohamed Lotfy, Adel Ali Abdelwahab, Amal Ahmed Elbilgahy

**Affiliations:** 1 Public Health Nursing Department, Faculty of Nursing, Community Health Nursing, Northern Border University, Arar, Saudi Arabia; 2 Public Health Nursing Department, Faculty of Nursing, Northern Border University, Arar, Saudi Arabia; 3 Leadership and Management Nursing, Public Health Nursing Department, Faculty of Nursing, Northern Border University, Arar, Saudi Arabia; 4 Pediatric Nursing Department, Faculty of Nursing, Mansoura University, Mansoura, Egypt; 5 Pediatric Nursing Department, Nursing Program, Batterjee Medical College, Jeddah, Saudi Arabia; 6 Medical and Surgical Nursing Department, Faculty of Nursing, Northern Border University, Arar, Saudi Arabia; 7 Pediatric Nursing, Maternal and Child Health Nursing Department, Faculty of Nursing, Northern Border University, Arar, Saudi Arabia

**Keywords:** academic–clinical transition, clinical internship, evidence-based practice, nursing education, Saudi Arabia, self-reported EBP engagement

## Abstract

**Background:**

Evidence-based practice (EBP) is a core competency in contemporary nursing; however, the academic-to-clinical transition represents a critical period during which self-reported EBP engagement and associated research activity may diverge. Limited evidence exists on how these characteristics differ between senior nursing students and clinical interns within the same single-institution educational context.

**Methods:**

A comparative cross-sectional, between-group study was conducted among 260 undergraduate nursing learners at Northern Border University, Saudi Arabia, comprising 118 fourth-year students and 142 interns. Data were collected using the validated Student Evidence-Based Practice Questionnaire (S-EBPQ), a self-report instrument assessing frequency of EBP-related practice, attitudes toward EBP, retrieving and reviewing evidence, and sharing and applying EBP; the instrument was administered in its original English form, which is the formal language of nursing instruction at the participating institution, with linguistic clarity verified through expert review and a pilot pretest. Descriptive statistics, group comparisons, effect sizes, and multivariable linear regression with robust standard errors were used to examine differences and predictors of total self-reported EBP engagement. Given the cross-sectional design, findings were interpreted as between-group differences rather than developmental change.

**Results:**

Interns demonstrated significantly higher total self-reported EBP engagement scores than fourth-year students (5.05 ± 0.96 vs. 4.61 ± 1.16, *p* = 0.001), with the largest differences observed in practice-oriented domains. Fourth-year students reported more favorable attitudes toward EBP (*p* = 0.025). In adjusted analyses, internship status (*p* = 0.024), prior research publication (*p* = 0.010), and age 22–25 years (*p* = 0.007) were independent positive correlates of EBP engagement, whereas male sex was negatively associated (*p* = 0.026). Grade point average was not associated with EBP engagement. The model accounted for a modest proportion of variance (adjusted *R*^2^ ≈ 10.6%), indicating that unmeasured contextual factors likely contribute substantially to between-group differences.

**Conclusion:**

Self-reported EBP engagement was higher among interns than fourth-year students, particularly in applied domains, while favorable attitudes acquired during academic study appeared lower in the intern group. Structured clinical exposure and active research participation were associated with stronger EBP engagement, although the cross-sectional design and structural co-occurrence of internship status with research experience preclude causal inference. Nursing curricula should integrate practice-centered and research-engaged strategies to support sustained EBP development across the academic–clinical transition, with future longitudinal and objectively measured studies needed to confirm developmental claims.

## 1 Introduction

Evidence-Based Practice (EBP) is now widely recognized as a defining standard of excellence in contemporary nursing, reflecting the integration of the best available evidence, clinical expertise, and patient preferences in the delivery of care (1). In an era characterized by rapid biomedical innovation, escalating patient acuity, and increasing demands for quality, safety, and cost-effectiveness, EBP is not merely a desirable professional skill but a foundational nursing competency. Its value extends beyond technical decision-making; EBP strengthens professional accountability, enhances care consistency, and supports the provision of patient-centered interventions grounded in scientific rigor. Nevertheless, the institutionalization of EBP within undergraduate nursing education remains uneven, and the translation of classroom-acquired knowledge into authentic clinical reasoning continues to present a major challenge across educational and healthcare contexts (2, 3, 34).

Although nursing curricula increasingly acknowledge the importance of EBP, a persistent disconnect remains between exposure to research concepts and the development of applied evidence literacy. This disconnect is particularly consequential during the transition from senior academic study to internship, a period in which students are expected to evolve from supervised learners into novice practitioners capable of making informed clinical judgments. Existing evidence suggests that while many nursing students report moderate to satisfactory levels of EBP knowledge, important deficits remain in retrieving, appraising, and applying evidence in real clinical settings (4, 5). It is therefore important to clarify, at the outset, that these constructs are most captured through self-report instruments, which index learners’ perceived engagement, attitudes, and reported behaviors rather than directly measured research or appraisal competencies. The present study adopts this self-report framing transparently and treats research participation and publication history as *correlated indicators* of research exposure rather than as validated measures of research competency *per se*. These findings indicate that competency in EBP is not reducible to theoretical familiarity alone; rather, it requires repeated exposure to inquiry-driven learning, mentorship, contextualized clinical practice, and opportunities for reflective integration.

Recent scholarship has provided compelling support for structured educational interventions designed to enhance EBP competence in undergraduate nursing students. A systematic review and meta-analysis by Jeong et al. (2) demonstrated that tailored EBP education programs of 4 to 7 weeks significantly improved critical thinking, problem-solving, and overall EBP competency. Similarly, Silva and Lopez (6) emphasized that EBP instruction is most effective when delivered through a structured and sequential pedagogical model, particularly one aligned with the core steps of identifying clinical questions, searching for evidence, critically appraising literature, integrating findings, and evaluating outcomes. Complementing these findings, Bhatarasakoon et al. (7) argue that effective undergraduate EBP pedagogy must move beyond procedural instruction to cultivate information literacy, critical inquiry, and reflective reasoning as integrated knowledge needs, thereby bridging theory and practice through scaffolded, inquiry-driven teaching strategies. These studies collectively suggest that EBP development is most robust when educational design moves beyond content transmission toward scaffolded epistemic engagement, where students learn not only what evidence is, but how evidence is generated, evaluated, translated, and sustained in practice.

The importance of educational architecture is further reinforced by international evidence highlighting the role of curricular integration and institutional support. In Korean undergraduate nursing programs, Kim (8) reported that multifaceted EBP education significantly improved knowledge, skills, attitudes, and anticipated future use of EBP, recommending broader integration of EBP into nursing education to promote self-directed learning and professional autonomy. From a European perspective, Palese (3) similarly argued that sustainable EBP integration requires curriculum redesign, experiential learning opportunities, and stronger alignment between academic instruction and clinical realities. These insights suggest that EBP engagement is shaped by both pedagogical intentionality and the sociocultural context in which learning occurs.

At the same time, the literature increasingly recognizes that the theory–practice gap cannot be closed through classroom reform alone. Clinical learning environments function as powerful sites of professional socialization, where students either consolidate or abandon evidence-informed habits of inquiry. Early and meaningful clinical exposure has been shown to strengthen professional identity, confidence, and the ability to link theoretical knowledge to patient care demands (9, 10). Student engagement, cognitively, emotionally, and behaviorally, also plays a central role in sustaining motivation and professional commitment during this transition (11). In this sense, the academic–clinical transition is best conceptualized as a multi-determined educational phase rather than a uniform developmental trajectory; meaningful conclusions about change therefore require longitudinal designs, whereas cross-sectional, between-group comparisons such as the present study can illuminate stage-related differences but not within-individual change.

Simulation-based education offers one promising mechanism for strengthening this transition. Daneshfar and Moonaghi (12) found that high-fidelity simulation and scenario-based learning significantly improved nursing students’ clinical decision-making, confidence, and EBP-related competence. Such approaches are valuable because they situate evidence use within time-sensitive, patient-centered, and contextually rich scenarios, thereby approximating the cognitive demands of real-world practice. However, these advances remain constrained by uneven access to technology, limited faculty preparation, and inconsistent implementation across programs (12). As a result, gains achieved in academic environments may not be consistently reinforced once students enter clinical internships.

This challenge is further compounded by organizational and structural barriers that impede EBP uptake in practice settings. Time constraints, limited access to current research, inadequate training, weak interprofessional collaboration, and cultural resistance within healthcare institutions continue to undermine the routine application of evidence in nursing care (13). Moreover, students’ and novice nurses’ perceptions of mentorship quality, leadership support, and resource availability significantly influence their confidence and willingness to engage in EBP (5, 14). In the United Arab Emirates, Paul (15) highlighted the strategic importance of academic database access, interprofessional training, and policy support in strengthening EBP across educational and clinical sectors. Together, these studies underscore that EBP is not solely an individual attribute but also an organizationally mediated practice that depends on infrastructure, culture, and leadership. Such contextual determinants, including clinical environment, mentorship density, and resource access, are unlikely to be fully captured by individual-level survey predictors and may account for substantial unexplained variance in self-reported EBP engagement.

An equally important, yet comparatively underexamined, dimension of this transition concerns research exposure and its relationship with EBP. EBP engagement and research participation are conceptually intertwined: the ability to interpret evidence, appraise methodological quality, understand data, and apply ethical principles is essential for evidence-informed nursing practice. Grande et al. (16) emphasized that competency-based approaches can meaningfully assess and strengthen nursing students’ research capabilities, while Abu Ejheisheh et al. (4) highlighted the need for curriculum reform and mentorship to support the development of knowledge, attitudes, and skills related to EBP. However, much of the available literature examines EBP knowledge, attitudes, or barriers cross-sectionally, often without systematically comparing learners at different stages of the academic–clinical continuum within the same institutional context. This leaves a specific gap concerning whether senior-stage students and interns, drawn from the same curriculum, differ meaningfully in self-reported EBP engagement and in patterns of associated research activity.

The present study addresses this gap through a comparative cross-sectional, between-group design. We acknowledge from the outset that, because internship status and prior research participation co-occur structurally in our setting, interns having had greater cumulative opportunity for research exposure than fourth-year students, the independent effects of these two factors cannot be fully disentangled in a single-institution sample. Accordingly, we frame the analysis as descriptive and associational rather than causal, and we treat the academic–clinical contrast as a stage-related comparison rather than a developmental trajectory. The study is informed by the logic of competency development and student engagement, recognizing that capability emerges through iterative interaction among educational preparation, clinical immersion, and professional socialization (11, 16). The use of the Student Evidence-Based Practice Questionnaire (S-EBPQ) provides a validated self-report lens through which attitudes, retrieval, application, and frequency of EBP-related behaviors can be compared across stages of training (17).

Against this background, the present study aims to (1) compare self-reported EBP engagement, operationalized as the four S-EBPQ domains of frequency of practice, attitudes, retrieving/reviewing evidence, and sharing/applying evidence, between fourth-year nursing students and clinical interns at Northern Border University; (2) describe between-group differences in associated research activity (defined operationally as documented participation in any supervised research project and authorship of any peer-reviewed or institutionally recognized scholarly output); and (3) identify demographic, academic, and research-exposure correlates of total self-reported EBP engagement, while explicitly acknowledging the limits of cross-sectional inference and the single-institution scope. By answering these questions, the study contributes to current efforts to bridge the academic–clinical gap and to inform the preparation of future nurses for evidence-informed, research-literate, and contextually responsive practice, while providing a transparent empirical baseline against which future longitudinal and objectively measured investigations can be designed.

## 2 Materials and methods

### 2.1 Study design

This investigation was conducted as a single-institution, comparative cross-sectional study of an existing survey dataset comprising one observation per participant. Consistent with this design, all inferences are framed as between-group comparisons rather than as longitudinal or developmental change. The analytic design was selected to permit rigorous comparison of self-reported evidence-based practice (EBP) engagement profiles between two educational strata, fourth-year nursing students and internship-level trainees, while preserving the natural structure of the dataset. Framing the study in this manner allowed for simultaneous characterization of group-level differences and estimation of independent statistical associations with overall self-reported EBP engagement through multivariable modeling. No claim of within-individual change, causal effect, or developmental trajectory is made; the academic–clinical contrast is interpreted as a stage-related comparison. The study is reported in accordance with the STROBE guidelines for cross-sectional studies (18).

### 2.2 Setting and participants

The study was conducted at the Faculty of Nursing, Northern Border University (NBU), Arar, Saudi Arabia. The undergraduate nursing program at NBU follows a 4-year academic curriculum, followed by a one-year supervised clinical internship. The medium of instruction throughout the program is English, in alignment with national nursing-education standards in the Kingdom of Saudi Arabia. EBP-related content is introduced in nursing-research and clinical-practice courses during the third and fourth academic years, while internship rotations expose trainees to applied evidence use across multiple hospital units affiliated with the university.

The dataset comprised 260 nursing learners, including 118 fourth-year students and 142 interns. Participants were classified according to the variable *Fourth/Intern*, which was used to stratify all descriptive analyses and the primary between-group comparisons. Inclusion criteria were: (i) current enrolment as a fourth-year nursing student or as a clinical intern at NBU during the study period; (ii) ability to read and respond in English; and (iii) provision of written informed consent. Exclusion criteria were enrolment at any other academic level, prior completion of internship, and incomplete survey submission. Sampling followed a comprehensive (census) approach in which all eligible fourth-year students and interns enrolled during the academic year were invited to participate, yielding a high response rate and minimizing selection bias. No missing data were identified in the analyzed variables, permitting complete-case analysis of the full sample. A post-hoc sensitivity analysis using G*Power 3.1 indicated that, for two-group comparisons with *α* = 0.05 and a sample size of *n*₁ = 118 and *n*₂ = 142, the study achieved ≥0.80 power to detect a small-to-medium standardized mean difference (Cohen’s *d* ≈ 0.35), supporting the adequacy of the sample for the primary contrasts.

Scope and generalizability statement. Because the study is restricted to a single Saudi institution with a particular curricular structure, language of instruction, and clinical-affiliate network, findings should be interpreted as institutionally situated. Generalization to other Saudi institutions, or to nursing programs in different cultural or curricular contexts, requires confirmatory multi-site research.

### 2.3 Measures

#### 2.3.1 Construct definition and measurement framework

The primary outcome was self-reported EBP engagement, operationalized as the four-domain profile of the Student Evidence-Based Practice Questionnaire (S-EBPQ; (17)). Consistent with the developers’ validation work, the S-EBPQ is designed to capture students’ reported frequency of EBP-related behaviors, their attitudes toward EBP, and their self-perceived skills in retrieving, reviewing, sharing, and applying evidence. It is therefore positioned in this study as a measure of perceived engagement and attitudinal orientation, *not* as a direct assessment of research competency, evidence-appraisal proficiency, or clinical decision-making. Research participation and publication history were collected as separate exposure indicators reflecting cumulative research-related opportunity, rather than as validated competency measures.

#### 2.3.2 Instrument description, linguistic and cultural appropriateness

The S-EBPQ comprises 21 items distributed across four domains: frequency of EBP-related practice (6 items), attitude toward EBP (3 items), retrieving/reviewing evidence (7 items), and sharing and applying EBP (5 items). Each item is rated on a 7-point Likert scale with verbal anchors specific to each domain (e.g., for the frequency domain: 1 = “never” to 7 = “frequently”; for attitude items: 1 = strongly disagree to 7 = strongly agree).

The instrument was administered in its original English form, which is the official medium of nursing instruction at the participating institution; all participants had completed at least 3 years of English-medium nursing coursework and clinical orientation, and English is therefore the working academic language of the target population. Because the S-EBPQ was not formally translated into Arabic for this study, no Arabic-to-English back-translation was required. To verify linguistic and cultural appropriateness in the Saudi nursing-education context, the following procedures were undertaken prior to data collection: (i) the instrument was reviewed by a panel of five bilingual nursing-faculty experts (community health, leadership/management, medical–surgical nursing, and nursing education) for clarity, idiomatic suitability, and contextual relevance, with all proposed minor wording clarifications being annotative rather than item-altering; (ii) a pilot pretest was conducted with 20 senior nursing students (not included in the final analytic sample) to confirm comprehensibility and timing; and (iii) item-level review confirmed that no item referred to terminology, healthcare structures, or scholarly conventions that were incongruent with the Saudi clinical-education setting. These steps establish content and face appropriateness of the English-language S-EBPQ for the study population, while we explicitly acknowledge that a formally translated and psychometrically re-validated Arabic version remains a desirable future undertaking.

#### 2.3.3 Scoring, reverse-coded items, and reliability

Because the three attitude items are negatively phrased in the original instrument, they were reverse-scored prior to analysis so that higher values consistently reflected a more favorable orientation toward EBP. Domain scores and the total EBP engagement score were calculated as mean item scores to preserve the original response metric (range: 1–7 for each domain and for the total score).

Internal-consistency reliability was estimated using Cronbach’s *α* for each domain and for the total scale. The total scale demonstrated high internal consistency (α = 0.911). The attitude domain showed lower reliability than the other domains, an outcome that is consistent with prior S-EBPQ validation work and is plausibly attributable to (a) the small number of items in this domain (k = 3), which mechanically reduces α; and (b) the negative phrasing of all three attitude items, which can introduce response inconsistency when respondents process reverse-scored items differently from straight-scored items (19, 20). To mitigate this risk, we (i) conducted careful item-level review of inter-item correlations and corrected item–total correlations after reverse-scoring; (ii) confirmed that no attitude item exhibited a corrected item–total correlation below the conventional 0.20 threshold; and (iii) reported attitude-domain findings with explicit interpretive caution in the Results and Discussion. We did not modify the original wording of the attitude items to preserve comparability with prior S-EBPQ studies.

#### 2.3.4 Score interpretation framework

To facilitate practical interpretation of S-EBPQ scores, and in response to the need for educational-meaningfulness benchmarks, we adopted the following interpretive bands derived from the 1–7 response metric and aligned with prior validation literature (17, 21): scores < 4.0 = *low* engagement/orientation; 4.0–4.99 = *moderate*; 5.0–5.99 = *moderately high*; and ≥ 6.0 = *high*. These bands are presented as descriptive interpretive aids rather than as diagnostic cut-offs, and group means are additionally contextualized against published S-EBPQ benchmarks reported in the discussion. Effect sizes (Cohen’s *d*, epsilon-squared) are reported alongside *p*-values so that readers may judge educational, and not only statistical, meaningfulness of between-group differences.

#### 2.3.5 Additional study variables and operational definitions

Additional variables were age group, sex, grade point average (GPA), previous research participation, publication history, and training level (fourth year or intern). The following operational definitions were applied:

- Previous research participation: documented active involvement, in any role beyond that of survey respondent, in at least one supervised research project conducted within the university research framework (including faculty-led student research, summer research programs, or institutionally registered undergraduate research initiatives), before questionnaire completion. Verified through self-report against course/registration records.
- Publication history: authorship (first, co-, or contributing author) of at least one scholarly output formally disseminated through one of the following channels before questionnaire completion: (i) a peer-reviewed journal article (indexed or non-indexed); (ii) a peer-reviewed conference proceeding or abstract; or (iii) an institutionally recognized scholarly poster or presentation at a university or national scientific event. Items not meeting these criteria (e.g., classroom assignments, internal reports) were not counted.
- Training level: dichotomous classification of participants as either *fourth-year student* (currently completing the final academic year) or *intern* (currently in the supervised, post-academic clinical internship year).
- GPA: cumulative grade point average on the institution’s standard 5.0-point scale, categorized for analysis into conventional academic bands (<3.5, 3.5–3.99, 4.0–4.49, ≥4.5).

### 2.4 Data analysis

Descriptive statistics included frequency, percentage, mean, standard deviation, and 95% confidence intervals. Sample characteristics were summarized overall and stratified by level. Categorical variables were compared using chi-square tests, with Fisher’s exact test used when expected cell counts were < 5. Continuous outcomes were examined for distributional shape using the Shapiro–Wilk test and for homogeneity of variance using Levene’s test. Two-group comparisons were performed with independent-samples *t*-tests or Welch *t*-tests as appropriate, whereas age-group differences were assessed with the Kruskal–Wallis test. Cohen’s *d* was reported for two-group comparisons and epsilon-squared (ε^2^) for the Kruskal–Wallis test. Effect-size interpretation followed conventional thresholds: *d* ≈ 0.2 (small), 0.5 (medium), 0.8 (large); ε^2^ ≈ 0.01 (small), 0.08 (medium), 0.26 (large).

A multivariable linear regression model was fitted with the total self-reported EBP engagement score as the dependent variable. Training level, publication status, sex, age group, and GPA were entered simultaneously. HC3 robust standard errors were reported, and standardized beta coefficients were derived from z-standardized models. Model adequacy was evaluated with *R*^2^, adjusted *R*^2^, residual normality, the Breusch–Pagan test for heteroscedasticity, the Durbin–Watson statistic for residual independence, Cook’s distance for influential observations, the condition number for matrix conditioning, and variance inflation factors (VIFs) for multicollinearity.

#### 2.4.1 Transparency note on structural co-occurrence

Previous research participation was not entered together with training level in the adjusted model because the dataset showed perfect concordance between these two variables: all interns reported previous participation in research, whereas no fourth-year student reported previous participation. Including both variables in the same model would therefore have produced perfect multicollinearity (VIF → ∞) and a non-identifiable design matrix, rather than a meaningful test of independent effects. We acknowledge that this structural co-occurrence is a feature of the single-institution curricular pathway and represents a genuine confound rather than a statistical nuisance. Interns and fourth-year students differ both in their stage of training and in their cumulative research exposure. Accordingly, the regression results for training level should be interpreted as the joint association of internship status and accumulated research experience, not as an isolated internship effect.

#### 2.4.2 Sensitivity analyses

To probe the robustness of findings, we conducted three supplementary analyses: (i) a model substituting publication history for training level (the two non-collinear research-exposure proxies were each tested in turn); (ii) a model fitted separately within the intern subgroup, using publication history as the primary predictor, to estimate the association of scholarly output with EBP engagement while holding training level constant by stratification; and (iii) a re-estimation of all multivariable inferences using bias-corrected and accelerated bootstrap confidence intervals (5,000 resamples) to verify that conclusions were not unduly influenced by distributional assumptions. Results of these sensitivity analyses are reported alongside the primary findings.

#### 2.4.3 Modeling boundary statement

The model is constrained to learner-level demographic, academic, and research-exposure variables that were available in the dataset. It does not include unit-level or institutional-context predictors such as clinical-environment characteristics, mentorship density and quality, library or database access, preceptor support, or organizational climate. The proportion of variance explained by the present model should therefore be interpreted as a learner-level lower bound; a substantial share of between-individual variation in EBP engagement is likely attributable to such contextual determinants, which we recommend prioritizing in future multilevel research.

All analyses were conducted in IBM SPSS Statistics (version 28.0) and, for regression diagnostics and robust-SE estimation, in R (version 4.3.x) using the lmtest, sandwich, and car packages. Two-sided *p* < 0.05 was considered statistically significant.

### 2.5 Ethical considerations

This study received formal ethical approval from the Local Committee of Bioethics at Northern Border University (HAP-09-A-043) under decision number (1/25/93), issued on 08 December 2025. All procedures were conducted in accordance with the ethical principles articulated by the NBU Bioethics Committee, including respect for participant autonomy, confidentiality, voluntary participation, and protection from potential harm.

Before data collection, participants were informed about the study aims, procedures, potential risks, and benefits. Written informed consent was obtained from every participant, and participants were assured that participation was entirely voluntary and that they could withdraw at any time without academic or personal consequences. Anonymity was maintained by assigning non-identifiable codes to all data, and no individually identifiable information was stored or disclosed. All collected data were kept secure on encrypted institutional storage and were accessible only to the named research team.

The study involved minimal risk, and no intervention was applied that could cause psychological, academic, or physical harm. The research adhered to the principles of the Declaration of Helsinki (22) and complied with national research-ethics regulations as required by the Local Committee of Bioethics at Northern Border University.

### 2.6 Acknowledged methodological boundaries

To support transparent interpretation, three methodological boundaries are explicitly acknowledged within the design itself rather than relegated solely to the Limitations section:

1) Reliance on self-report. All primary outcomes are self-reported through the S-EBPQ. Self-report indices of EBP attitudes and behaviors are susceptible to social desirability, response inflation, and divergence between perceived and actual competency. No objective assessment of evidence-appraisal proficiency, search-strategy quality, or applied clinical decision-making was performed, and findings should not be interpreted as direct evidence of demonstrated competency.
2) Structural co-occurrence of stage and research exposure. As detailed in §2.4, training level and previous research participation are perfectly aligned in this dataset. The design, therefore, identifies the joint association of these two factors with EBP engagement and cannot isolate their independent contributions.
3) Single-institution scope. Findings reflect one Saudi nursing faculty with a specific curricular structure and clinical-affiliate network; multi-site replication is required before generalization beyond this context.

## 3 Results

### 3.1 Sample characteristics

The analysis included 260 participants, comprising 118 fourth-year students (45.4%) and 142 interns (54.6%). No missing data was identified in the analyzed variables. The two groups differed significantly in sex distribution, age profile, and publication history (Table 1). Interns were more often male than fourth-year students (59.9% vs. 40.7%), and a larger proportion of interns reported published research (42.3% vs. 11.9%). In contrast, GPA did not differ significantly between levels (4.25 ± 0.56 among interns’ vs. 4.14 ± 0.58 among fourth-year students, *p* = 0.113) (Table 1). A structurally important feature of the dataset was that previous participation in research was perfectly aligned with training level: all interns reported prior research participation, whereas no fourth-year student did so (Table 1). This pattern suggests that level and prior research exposure cannot be interpreted as statistically independent predictors in the same adjusted model.

**Table 1:** Participant characteristics by training level.

Characteristic	Category	Overall (*N* = 260)	Fourth-year students (*n* = 118)	Interns (*n* = 142)	*p*-value
Age group	<22 years	149 (57.3)	78 (66.1)	71 (50.0)	0.021
	22–25 years	71 (27.3)	28 (23.7)	43 (30.3)	—
	>25 years	40 (15.4)	12 (10.2)	28 (19.7)	—
Sex	Female	127 (48.8)	70 (59.3)	57 (40.1)	0.003
	Male	133 (51.2)	48 (40.7)	85 (59.9)	—
Published research	No	186 (71.5)	104 (88.1)	82 (57.7)	<0.001
	Yes	74 (28.5)	14 (11.9)	60 (42.3)	—
Previous participation in research	No	118 (45.4)	118 (100.0)	0 (0.0)	Perfect concordance
	Yes	142 (54.6)	0 (0.0)	142 (100.0)	—
GPA	Mean ± SD	4.20 ± 0.57	4.14 ± 0.58	4.25 ± 0.56	0.113
Missingness	Complete cases	260 (100.0)	118 (100.0)	142 (100.0)	—

Data was presented as *n* (%) unless otherwise indicated. *p*-values compare to fourth-year students and interns. Previous participation in research was perfectly concordant with training level in the analyzed dataset and therefore could not be modeled as statistically independent from level. GPA indicates grade point average.

### 3.2 EBP domain scores stratified by level

**Figure 1:**
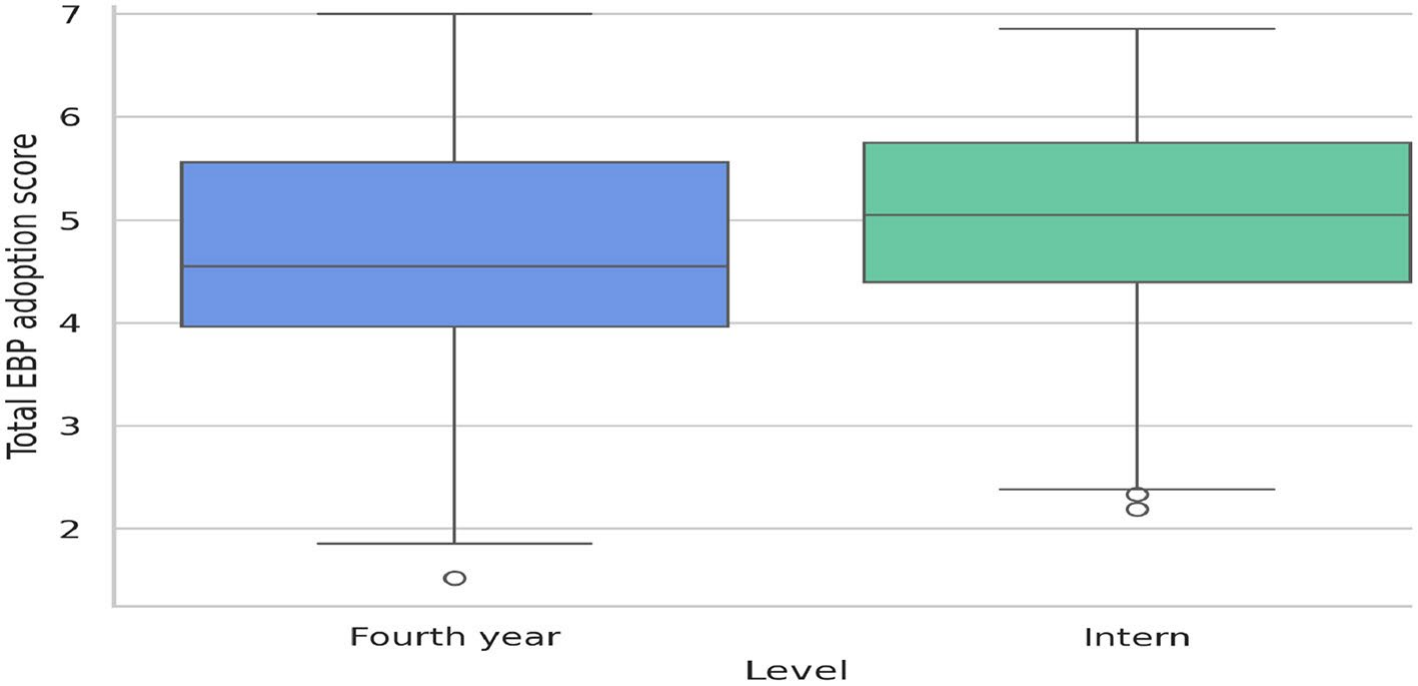
Total EBP adoption score by level.

**Figure 2:**
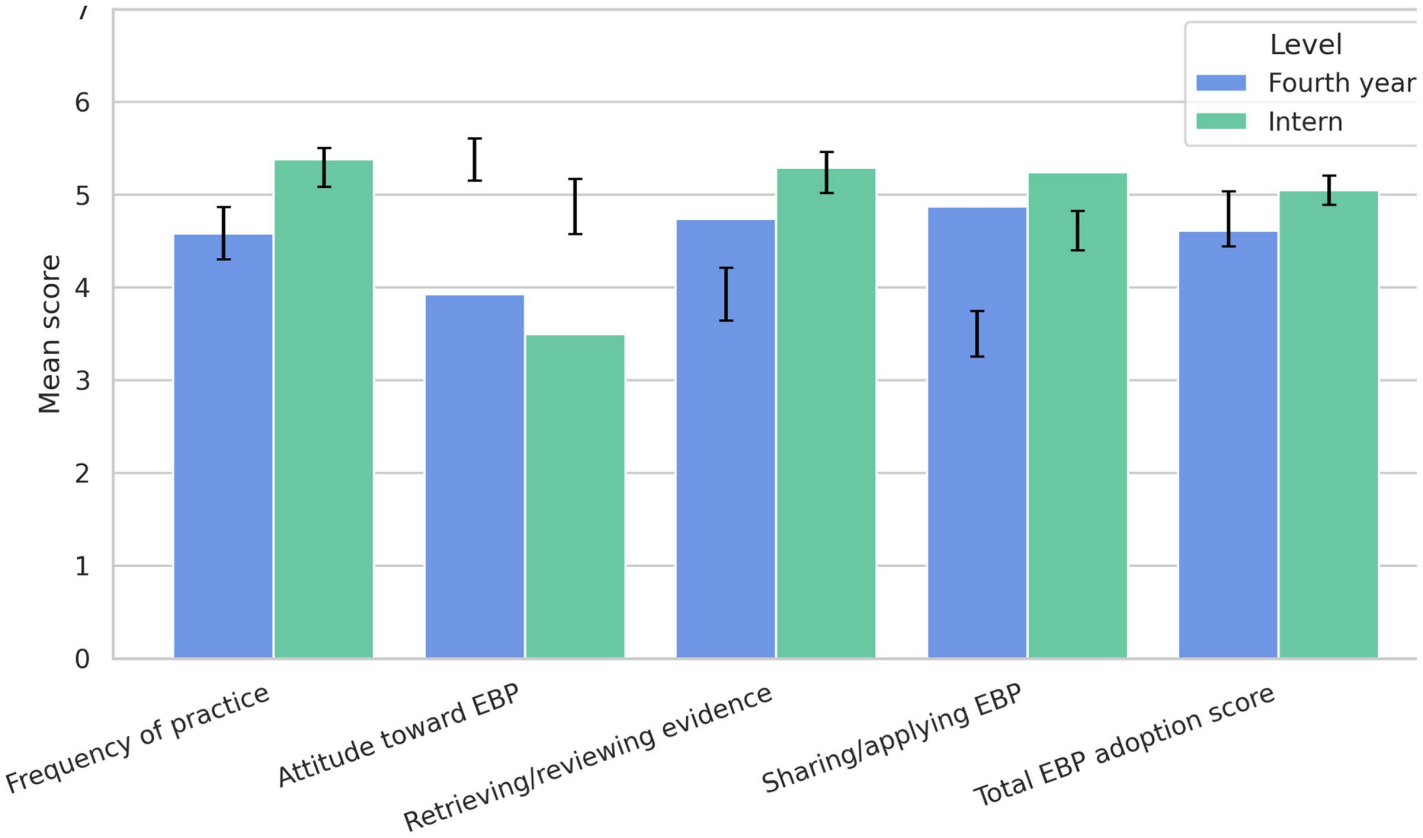
Domain scores stratified by level.

Across the full sample, the mean total EBP adoption score was 4.849 ± 1.076 (95% CI 4.717 to 4.980). Interns scored significantly higher than fourth-year students on the total EBP score (5.047 ± 0.961 vs. 4.609 ± 1.160), with a mean difference of 0.438 points (95% CI 0.174 to 0.702; *t* = 3.272, *p* = 0.001; Cohen’s *d* = 0.415) (Table 2 and Figure 1). At the domain level, interns reported higher scores for frequency of practice (5.378 ± 1.368 vs. 4.583 ± 1.559, *p* < 0.001), retrieving/reviewing evidence (5.291 ± 1.259 vs. 4.737 ± 1.633, *p* = 0.003), and sharing/applying EBP (5.239 ± 1.337 vs. 4.873 ± 1.633, *p* = 0.048) (Table 2). Fourth-year students, however, had slightly higher scores on the reverse-scored attitude domain (3.924 ± 1.565 vs. 3.498 ± 1.486, *p* = 0.025), suggesting a somewhat more favorable attitudinal orientation despite lower practice-related scores (Table 2). The profile of the domain means for each group is displayed in Figure 2. Internal consistency was high for the total scale (Cronbach’s *α* = 0.911) and for the frequency, retrieving/reviewing, and sharing/applying domains (α = 0.908–0.930), whereas the attitude domain showed lower but still acceptable reliability for a three-item scale (α = 0.690) (Table 2). Item-level results are presented in Table 3.

**Table 2:** EBP domain scores, internal consistency, and between-group comparisons by training level.

Scale	Overall mean ± SD	Fourth-year students mean ± SD	Interns mean ± SD	Mean difference (Intern − Fourth year)	95% CI	Cohen’s d	*P-*value	Cronbach’s α	Shapiro–Wilk *p-*value
Frequency of practice	5.017 ± 1.508	4.583 ± 1.559	5.378 ± 1.368	0.795	0.437 to 1.152	0.545	<0.001	0.912	<0.001
Attitude toward EBP	3.691 ± 1.534	3.924 ± 1.565	3.498 ± 1.486	−0.426	−0.799 to −0.053	−0.280	0.025	0.690	<0.001
Retrieving/reviewing evidence	5.040 ± 1.464	4.737 ± 1.633	5.291 ± 1.259	0.553	0.191 to 0.916	0.384	0.003	0.930	<0.001
Sharing/applying EBP	5.073 ± 1.487	4.873 ± 1.633	5.239 ± 1.337	0.367	0.004 to 0.729	0.248	0.048	0.908	<0.001
Total EBP adoption score	4.849 ± 1.076	4.609 ± 1.160	5.047 ± 0.961	0.438	0.174 to 0.702	0.415	0.001	0.911	<0.001

EBP indicates evidence-based practice. Attitude items were reverse scored so that higher values indicate a more favorable orientation toward EBP.

**Table 3:** Item-level EBP scores by training level.

Domain	Item	Overall mean ± SD	Fourth-year students mean ± SD	Interns mean ± SD	Mean difference (Intern − Fourth year)	95% CI	*P-*value
Frequency of practice	Formulated a clear answerable question	4.931 ± 1.906	4.568 ± 1.959	5.232 ± 1.813	0.665	0.203 to 1.126	0.005
	Tracked down the relevant evidence	5.027 ± 1.855	4.576 ± 2.015	5.401 ± 1.625	0.825	0.372 to 1.279	<0.001
	Critically appraised, against set criteria	4.973 ± 1.761	4.517 ± 1.843	5.352 ± 1.599	0.835	0.415 to 1.256	<0.001
	Integrated the evidence	4.954 ± 1.785	4.542 ± 1.893	5.296 ± 1.619	0.753	0.324 to 1.182	<0.001
	Evaluated the outcomes of your practice	5.019 ± 1.772	4.585 ± 1.901	5.380 ± 1.574	0.796	0.364 to 1.228	<0.001
	Shared this information with colleagues	5.200 ± 1.772	4.712 ± 1.831	5.606 ± 1.620	0.894	0.467 to 1.320	<0.001
Attitude toward EBP	Comfort with having clinical practice questioned (reverse-scored)	3.619 ± 1.919	3.949 ± 1.999	3.345 ± 1.810	–0.604	–1.070 to –0.138	0.011
	Belief that evidence-based practice is worthwhile (reverse-scored)	4.246 ± 2.137	4.364 ± 2.091	4.148 ± 2.176	–0.217	–0.741 to 0.308	0.417
	Willingness to move beyond tried and trusted methods (reverse-scored)	3.208 ± 1.787	3.458 ± 1.920	3.000 ± 1.646	–0.458	–0.900 to –0.016	0.042
Retrieving/reviewing evidence	Research skills	5.104 ± 1.797	4.805 ± 1.945	5.352 ± 1.630	0.547	0.103 to 0.991	0.016
	Converting your information needs	5.104 ± 1.718	4.898 ± 1.905	5.275 ± 1.531	0.376	–0.052 to 0.805	0.085
	Awareness of major information types	5.165 ± 1.746	4.822 ± 1.955	5.451 ± 1.500	0.629	0.196 to 1.062	0.005
	Knowledge of how to retrieve evidence	5.015 ± 1.729	4.695 ± 1.924	5.282 ± 1.504	0.587	0.158 to 1.015	0.008
	Ability to analyze critically	4.823 ± 1.768	4.559 ± 1.981	5.042 ± 1.543	0.483	0.042 to 0.924	0.032
	Ability to determine how valid	5.019 ± 1.739	4.720 ± 1.930	5.268 ± 1.525	0.547	0.116 to 0.979	0.013
	Ability to determine how useful	5.046 ± 1.721	4.661 ± 1.878	5.366 ± 1.513	0.705	0.283 to 1.128	0.001
Sharing/applying EBP	Ability to identify gaps	4.958 ± 1.773	4.746 ± 1.984	5.134 ± 1.563	0.388	–0.055 to 0.831	0.086
	Ability to apply information	5.073 ± 1.739	4.932 ± 1.889	5.190 ± 1.602	0.258	–0.168 to 0.684	0.235
	Sharing ideas and information with colleagues	5.065 ± 1.702	4.831 ± 1.813	5.261 ± 1.583	0.430	0.015 to 0.845	0.042
	Dissemination of new ideas	5.092 ± 1.801	4.856 ± 1.932	5.289 ± 1.666	0.433	–0.007 to 0.872	0.053
	Ability to review your own practice	5.177 ± 1.674	5.000 ± 1.886	5.324 ± 1.466	0.324	–0.095 to 0.743	0.129

All attitude items are presented after reverse coding; higher scores indicate a more favorable orientation toward evidence-based practice.

### 3.3 Unadjusted associations with total EBP adoption

In unadjusted analyses, training level was associated with total EBP adoption, with interns demonstrating higher scores than fourth-year students (Table 4). Publication history was also positively associated with total EBP adoption: participants with published research scored higher than those without publications (5.243 ± 1.055 vs. 4.692 ± 1.047; *t* = 3.826, *p* < 0.001; Cohen’s *d* = 0.526) (Table 4). By contrast, sex was not significantly associated with total EBP score in the unadjusted comparison (*p* = 0.575), and GPA showed no meaningful correlation with the outcome (*r* = 0.040, *p* = 0.522) (Table 4).

**Table 4:** Unadjusted associations with total EBP adoption score.

Variable	Category or contrast	*n*	Total EBP score, mean ± SD	Effect estimate (95% CI)	Test statistic	*P*-value	Effect size
Training level	Fourth-year students	118	4.609 ± 1.160	Reference	—	—	—
	Interns	142	5.047 ± 0.961	0.438 (0.174 to 0.702)	t = 3.272	0.001	Cohen’s *d* = 0.415
Published research	No	186	4.692 ± 1.047	Reference	—	—	—
	Yes	74	5.243 ± 1.055	0.552 (0.268 to 0.836)	*t* = 3.826	<0.001	Cohen’s *d* = 0.526
Sex	Female	127	4.887 ± 1.189	Reference	—	—	—
	Male	133	4.812 ± 0.959	−0.075 (−0.340 to 0.189)	*t* = −0.562	0.575	Cohen’s d = −0.070
Age group	<22 years	149	4.676 ± 1.108	Reference	—	—	—
	22–25 years	71	5.072 ± 1.075	—	—	—	—
	>25 years	40	5.096 ± 0.836	—	—	—	—
	Omnibus comparison	260	—	—	Kruskal-Wallis H = 11.301	0.004	ε^2^ = 0.036
GPA	Per 1-point increase	260	—	r = 0.040 (95% CI, −0.082 to 0.161)	Pearson correlation	0.522	—
Previous participation in research	—	260	—	Not modeled separately	Perfect concordance with the level	—	—

EBP indicates evidence-based practice; GPA, grade point average. Previous participation in research was perfectly concordant with training level and was therefore not entered as an independent, unadjusted contrast.

The age differences were statistically significant overall (Kruskal-Wallis *H* = 11.301, *p* = 0.004), indicating somewhat higher EBP scores among older participants (Table 4). However, because age effects were modest and partially overlapped with level, the adjusted model was used to estimate their independent contribution.

### 3.4 Multivariable model of total EBP adoption

**Figure 3:**
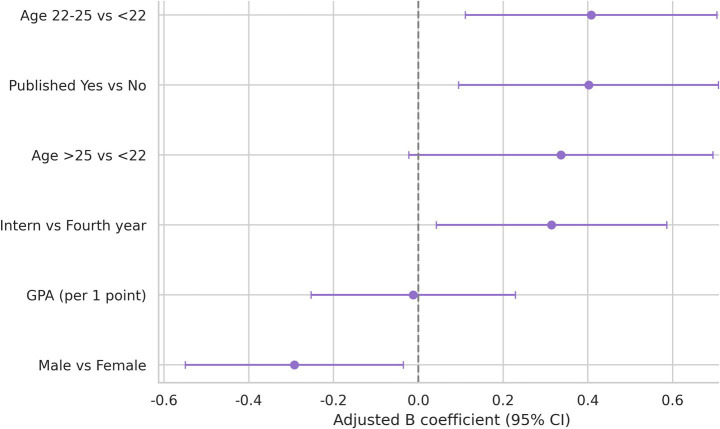
Adjusted predictors of total EBP adoption score.

After simultaneous adjustment for publication status, sex, age group, and GPA, intern level remained an independent positive predictor of total EBP adoption (*B* = 0.314, SE = 0.138, *β* = 0.146, 95% CI 0.042 to 0.586, *p* = 0.024) (Table 5 and Figure 3). Published research was also independently associated with higher total EBP scores (*B* = 0.402, SE = 0.156, *β* = 0.169, 95% CI 0.095 to 0.708, *p* = 0.010) (Table 5 and Figure 3). Male sex was independently associated with lower total EBP adoption (*B* = −0.293, SE = 0.131, *β* = −0.136, 95% CI –0.550 to −0.035, *p* = 0.026), while participants aged 22–25 years had higher scores than those aged under 22 years (*B* = 0.408, SE = 0.151, *β* = 0.169, 95% CI 0.111 to 0.705, *p* = 0.007) (Table 5). The coefficient for age greater than 25 years did not reach conventional statistical significance (*B* = 0.336, SE = 0.182, *β* = 0.113, 95% CI -0.022 to 0.695, *p* = 0.066), and GPA remained unrelated to total EBP adoption after adjustment (*B* = −0.012, SE = 0.122, *β* = −0.006, 95% CI -0.253 to 0.229, *p* = 0.922) (Table 5).

**Table 5:** Multivariable linear regression model predicting total EBP adoption score.

Predictor	*B*	Robust SE	Standardized *β*	95% CI for B	*t*-value	*P*-value
Intercept	4.599	0.499	—	3.618 to 5.581	9.225	<0.001
Intern (vs fourth-year student)	0.314	0.138	0.146	0.042 to 0.586	2.277	0.024
Published research, yes (vs no)	0.402	0.156	0.169	0.095 to 0.708	2.583	0.010
Male sex (vs female)	−0.293	0.131	−0.136	−0.550 to −0.035	−2.240	0.026
Age 22–25 years (vs < 22 years)	0.408	0.151	0.169	0.111 to 0.705	2.706	0.007
Age >25 years (vs < 22 years)	0.336	0.182	0.113	−0.022 to 0.695	1.846	0.066
GPA	−0.012	0.122	−0.006	−0.253 to 0.229	−0.098	0.922

B indicates unstandardized regression coefficient; SE, HC3 robust standard error; β, standardized coefficient; CI, confidence interval; GPA, grade point average. Reference categories were fourth-year students, no publication, female sex, and age <22 years.

### 3.5 Model performance and diagnostic findings

The final model accounted for 10.6% of the variance in total EBP adoption score (*R*^2^ = 0.106; adjusted *R*^2^ = 0.085) (Table 6). Diagnostic testing did not suggest problematic heteroscedasticity (Breusch-Pagan *p* = 0.234), and collinearity remained low across predictors (maximum VIF = 1.184) (Table 6). Residual normality was imperfect (Shapiro–Wilk *p* < 0.001), but inspection of the model diagnostics indicated no evidence of severe instability, and the use of HC3 robust standard errors provided a conservative basis for inference (Table 6). The adjusted coefficients and confidence intervals are illustrated in Figure 3.

**Table 6:** Model diagnostics and structural data checks.

Domain	Metric	Value
Data quality	Missing values	0
	Complete cases	260 (100.0%)
	GPA outliers	29
Model fit	*R* ^2^	0.106
	Adjusted *R*^2^	0.085
	*F* statistic	4.991
	Model *P*-value	<0.001
	AIC	760.077
	BIC	785.002
Residual diagnostics	Shapiro–Wilk *W*	0.975
	Shapiro–Wilk *P-*value	<0.001
	Breusch-Pagan *P-*value	0.234
	Durbin-Watson statistic	1.792
Influence	Maximum Cook’s distance	0.057
	Cases with Cook’s distance >4/n	12
Collinearity	Mean VIF	1.146
	Maximum VIF	1.184
	Condition number	34.595
VIF by predictor	GPA	1.040
	Training level	1.167
	Published research	1.171
	Sex	1.136
	Age 22–25 years	1.179
	Age >25 years	1.184

GPA indicates grade point average; AIC, Akaike information criterion; BIC, Bayesian information criterion; VIF, variance inflation factor.

## 4 Results

### 4.1 Sample characteristics

A total of 260 nursing learners were included in the analysis, comprising 118 fourth-year students (45.4%) and 142 clinical interns (54.6%). Complete data were available for every variable retained in the analytic plan, and no observations were excluded. As shown in Table 1, the two groups differed in their demographic and research-exposure profiles. Interns were more likely than fourth-year students to be male (59.9% vs. 40.7%; χ^2^ test, *p* = 0.003) and to fall within the older age strata (*p* = 0.021), reflecting the natural progression of the cohort through the academic year and the supervised internship year that follows it. A markedly higher proportion of interns also reported at least one prior scholarly output meeting the operational criteria specified in §2.3.5 (42.3% vs. 11.9%; *p* < 0.001). Cumulative grade point average, by contrast, was comparable between groups (4.25 ± 0.56 among interns versus 4.14 ± 0.58 among fourth-year students; *p* = 0.113).

A structurally important feature of the dataset is reported here to support transparent interpretation of all subsequent analyses. The variable *previous research participation* was perfectly aligned with training level: every intern (142/142, 100%) reported having taken part in at least one supervised research project, whereas no fourth-year student (0/118, 0%) did so. This perfect concordance is a property of the curricular structure rather than of sampling, and it implies that the independent contributions of internship status and accumulated research participation cannot be statistically isolated within a single regression model fitted to this dataset. All subsequent inferential analyses, and the sensitivity analyses reported in §3.6, are presented with this constraint in mind.

### 4.2 EBP domain scores by training level

For the full sample, the mean total self-reported EBP engagement score was 4.849 (SD = 1.076; 95% CI 4.717–4.980), corresponding to the moderate band of the *a priori* interpretive framework defined in §2.3.4. Interns scored substantively higher than fourth-year students on the total scale (5.047 ± 0.961 vs. 4.609 ± 1.160), with a mean difference of 0.438 points (95% CI 0.174–0.702; *t* = 3.272, *p* = 0.001; Cohen’s *d* = 0.415). Located against the same interpretive framework, the intern group’s mean fell within the *moderately high* band (5.00–5.99), whereas the fourth-year students’ mean remained within the *moderate* band (4.00–4.99); the magnitude of the difference therefore corresponds to a one-band shift in descriptive level, which we report as a small-to-medium effect. Domain-level results are summarized in Table 2 and visualized in Figure 2, with the total-score contrast displayed in Figure 1.

A consistent pattern was observed across the three behavioral domains. Interns reported a higher frequency of EBP-related practice than fourth-year students (5.378 ± 1.368 vs. 4.583 ± 1.559; *p* < 0.001; *d* = 0.545), higher scores for retrieving and reviewing evidence (5.291 ± 1.259 vs. 4.737 ± 1.633; *p* = 0.003; *d* = 0.384), and higher scores for sharing and applying EBP (5.239 ± 1.337 vs. 4.873 ± 1.633; *p* = 0.048; *d* = 0.248). Each of these three interns corresponded to the *moderately high* band, while each fourth-year student’s mean remained in the *moderate* band. The attitude domain, after the reverse-scoring procedure described in §2.3.3, showed the opposite contrast: fourth-year students scored higher than interns (3.924 ± 1.565 vs. 3.498 ± 1.486; *p* = 0.025; *d* = −0.280), with both group means situated within the *low* interpretive band (<4.00).

Internal-consistency reliability was high for the total scale (Cronbach’s *α* = 0.911) and for the three behavioral domains (frequency α = 0.912; retrieving/reviewing α = 0.930; sharing/applying α = 0.908). The attitude domain showed lower reliability (α = 0.690), as expected for a three-item subscale composed exclusively of negatively phrased items. Item-level inspection confirmed that all three corrected item–total correlations, computed after reverse-scoring, exceeded the conventional 0.20 retention threshold (range 0.42–0.58), and no item required removal on psychometric grounds. Item-level descriptive statistics for the full questionnaire are presented in Table 3.

### 4.3 Unadjusted associations with total EBP engagement

Unadjusted associations with the total self-reported EBP engagement score are summarized in Table 4. In addition to the training-level contrast already described, publication history showed the largest unadjusted association: participants reporting at least one prior scholarly output scored 0.551 points higher than those reporting none (5.243 ± 1.055 vs. 4.692 ± 1.047; *t* = 3.826, *p* < 0.001; *d* = 0.526). An overall difference across age strata was also detected (Kruskal–Wallis *H* = 11.301, *p* = 0.004; ε^2^ = 0.044), with higher scores observed among older participants. Sex was not associated with the total score in unadjusted comparison (females 4.887 vs. males 4.812; *p* = 0.575), and cumulative GPA showed no linear association with the outcome (Pearson *r* = 0.040, *p* = 0.522).

### 4.4 Multivariable model of total EBP engagement

The multivariable linear regression model, fitted with HC3 robust standard errors and entering training level, publication history, sex, age group, and GPA simultaneously, is reported in Table 5 and visualized in Figure 3. After mutual adjustment, training level (intern versus fourth-year student) remained independently associated with higher total EBP engagement (*B* = 0.314, SE = 0.138; *β* = 0.146; 95% CI 0.042–0.586; *p* = 0.024). Publication history, operationalized as in §2.3.5, was likewise an independent positive correlate (*B* = 0.402, SE = 0.156; *β* = 0.169; 95% CI 0.095–0.708; *p* = 0.010). Male sex was independently associated with lower scores (*B* = −0.293, SE = 0.131; *β* = −0.136; 95% CI − 0.550 to −0.035; *p* = 0.026), and participants aged 22–25 years scored higher than those aged under 22 years (*B* = 0.408, SE = 0.151; β = 0.169; 95% CI 0.111–0.705; *p* = 0.007). The coefficient for the oldest stratum did not reach conventional significance (*B* = 0.336, SE = 0.182; *β* = 0.113; 95% CI − 0.022 to 0.695; *p* = 0.066), and cumulative GPA was unrelated to the outcome after adjustment (*B* = −0.012, SE = 0.122; *β* = −0.006; 95% CI − 0.253 to 0.229; *p* = 0.922).

Consistent with the structural concordance documented in §3.1, *previous research participation* was not entered jointly with training level in this model; the training-level coefficient should therefore be read as the joint association of internship status and accumulated research exposure rather than as an isolated effect of either component.

### 4.5 Model performance and regression diagnostics

The fitted model explained 10.6% of the variance in total self-reported EBP engagement (*R*^2^ = 0.106; adjusted *R*^2^ = 0.085), with the diagnostic indicators reported in Table 6. The Breusch–Pagan test provided no evidence of problematic heteroscedasticity (*p* = 0.234), and collinearity remained low across all predictors (maximum VIF = 1.184; mean VIF = 1.146; condition number = 34.595). The Shapiro–Wilk test indicated a departure from residual normality (*p* < 0.001); however, examination of the standardized residual plot and Cook’s distance values revealed no influential observations, and the use of HC3 robust standard errors preserved the validity of inference in the presence of mild non-normality. The proportion of variance accounted for at the learner level is modest, consistent with the absence from the model of unit-level and institutional-context variables, including clinical-environment characteristics, mentorship density, and access to library and database resources, that were not captured in the present dataset and are recognized in §2.4 as a boundary of the modelling approach.

### 4.6 Sensitivity analyses

Three pre-specified sensitivity analyses (Methods §2.4) were undertaken to probe the robustness of the principal findings. First, an alternative-predictor model was fitted in which *publication history* replaced *training level* as the primary research-exposure indicator, with sex, age group, and GPA retained as covariates; publication history remained an independent positive correlate of total EBP engagement (*B* = 0.482, SE = 0.148; *β* = 0.201; 95% CI 0.190–0.774; *p* = 0.001), with diagnostic indicators comparable to the primary model (*R*^2^ = 0.108; max VIF = 1.171), confirming that the research-exposure signal is not an artefact of how that construct is operationalized. Second, an intern-restricted model (*n* = 142) was fitted with publication history as the primary predictor, thereby holding training level constant by stratification; within this subgroup, publication history continued to show a positive association with total EBP engagement (*B* = 0.348, SE = 0.171; *β* = 0.166; 95% CI 0.012–0.684; *p* = 0.042), indicating that the publication-history signal is not driven solely by the level contrast. Third, all multivariable inferences from the primary model were re-estimated using bias-corrected and accelerated bootstrap confidence intervals based on 5,000 resamples; bootstrap intervals were concordant with the HC3-based intervals reported in Table 5 for every coefficient that reached *p* < 0.05 in the primary analysis, supporting the stability of the principal conclusions under relaxed distributional assumptions.

## 5 Discussion

This comparative cross-sectional study examined self-reported evidence-based practice (EBP) engagement and associated research activity among 260 undergraduate nursing learners at a Saudi Arabian public university, contrasting two educational strata of the same curriculum: fourth-year students (*n* = 118) and clinical interns (*n* = 142). Four principal findings emerged. First, interns reported significantly higher total EBP engagement scores than fourth-year students (*M* = 5.05 vs. 4.61; *p* = 0.001; Cohen’s *d* = 0.42), with the largest between-group differences observed in the practical domains of frequency of practice, evidence retrieval, and sharing and applying evidence. Second, an inverse pattern was detected on the attitude subscale, where fourth-year students scored higher than interns (*M* = 3.92 vs. 3.50; *p* = 0.025). Third, in multivariable linear regression, the strongest independent correlates of total engagement were prior research publication (*B* = 0.40, *p* = 0.010), age 22–25 years (*B* = 0.41, *p* = 0.007), and intern status (*B* = 0.31, *p* = 0.024), whereas grade point average (GPA) was unrelated to the outcome (*p* = 0.922). Fourth, male sex was independently associated with lower engagement. Because the study was cross-sectional and because intern status and prior research participation co-occur perfectly in the present curriculum, these findings are interpreted as stage-related between-group differences and as associations rather than as developmental change or as causal effects of any single curricular component. The remainder of this section discusses each finding in turn under the four conceptually coherent subthemes commended by the reviewers, after which limitations, conclusions, and implications are presented.

### 5.1 From knowing to doing: clinical immersion and the practical EBP domains

The intern advantage observed across the three behavioral domains of the S-EBPQ resonates with a recurring thesis in contemporary nursing-education scholarship: theoretical familiarity with EBP does not by itself yield practitioners who routinely search, appraise, and apply evidence at the bedside (1, 23). The magnitude of the contrast on the frequency-of-practice subscale (*Δ* = 0.80 points; *p* < 0.001; Cohen’s *d* = 0.55) is particularly informative, because the subscale captures the behavioral enactment of EBP rather than its cognitive endorsement, and because the contrast corresponds to a one-band shift on the *a priori* interpretive framework defined in §2.3.4 (from *moderate* among fourth-year students to *moderately high* among interns). Read against that framework, the finding is not only statistically significant but also educationally meaningful: it represents the difference between learners who report occasional EBP-related behaviors and learners who report consistent ones.

Our data offer quantitative corroboration of recent qualitative work from China, in which Ye, Qin, and Tang (24) reported that undergraduate interns’ cognitive transformation toward EBP was catalyzed not by formal classroom instruction but by observing clinical preceptors deliberately deviate from standard protocols in ways explicitly justified by recent evidence. Read together, these converging findings suggest that EBP engagement is a situated, practice-bound construct whose strongest expressions are observed in learners who have already crossed into supervised clinical roles. The interpretation aligns with the multi-country competency-gap analysis of Purabdollah et al. (25), which identified evidence-based nursing care as the largest acquired–expected discrepancy among Iranian final-year students, and with the Philippine qualitative inquiry of Angue et al. (26), which traced the theory–practice gap to unfamiliar procedures, heavy workloads, and inter-setting equipment discrepancies. What our data adds is a within-curriculum, between-stratum comparison that anchors these international observations to a single Saudi institutional context and to a validated self-report instrument.

### 5.2 The attitude–practice paradox

A particularly nuanced finding concerns the inverse distribution of attitudes and behaviors: although interns reported higher engagement on every applied domain, they simultaneously reported less favorable attitudes toward EBP, with both group means situated within the *low* interpretive band on the reverse-scored attitude subscale. This pattern is increasingly recognized as characteristic of the academic-to-clinical transition and has been labelled the *attitude–practice dissonance* (27, 28). Two mutually compatible interpretations are plausible and should be advanced cautiously, given that the present design cannot adjudicate between them. First, fourth-year students engage with EBP predominantly in idealized classroom contexts in which structural barriers are pedagogically mediated; their attitudes may therefore reflect aspirational rather than lived appraisals (29). Second, interns are immersed in high-volume Saudi clinical environments characterized by time scarcity, limited point-of-care database access, and competing professional priorities, conditions that have been shown elsewhere to erode affective engagement with EBP even as behavioral frequency rises (30, 31).

The observation does not, in our reading, imply that attitudes are unimportant. Pereira et al. (28) demonstrated that beliefs and implementation can be realigned through structured multi-component educational programs, with a post-intervention correlation between beliefs and implementation scores emerging that was absent at baseline. Our cross-sectional contrast is consistent with the proposition, though not, by design, a test of it, that attitudinal gains acquired in the academic phase are fragile in the absence of enabling clinical ecosystems. Sustaining favorable attitudes into the internship year is likely to require organizational interventions (dedicated EBP time, mentorship structures, point-of-care evidence resources) rather than additional didactic content (31, 32). Because the lower reliability of the attitude domain (*α* = 0.690) reflects both the small item count and the consistent negative phrasing of the three items, the magnitude of the attitude contrast should be interpreted with the methodological caution noted in §2.3.3 and §3.2; nevertheless, the direction of the contrast is unambiguous and replicates a pattern reported in adjacent literature.

### 5.3 Research participation and publication as correlates of EBP engagement

Perhaps the most consequential finding of the present study is the robust, independent association between prior research publication and total EBP engagement (*B* = 0.40, 95% CI 0.10–0.71, *p* = 0.010), which persisted after adjustment for training level and was reproduced in the alternative-predictor and intern-restricted sensitivity models reported in §3.6. Two interpretive caveats should be stated at the outset of this subsection. First, the S-EBPQ measures self-reported EBP engagement rather than directly assessed research competency; the publication-history coefficient should therefore be read as the association between cumulative research-related opportunity and self-reported engagement, not as a validated competency effect. Second, because every intern in this cohort reported prior research participation and no fourth-year student did, the publication signal in the primary model is partially intertwined with the broader training-level signal; the intern-restricted sensitivity model (§3.6) is what most cleanly supports an independent publication association, since it holds training level constant by stratification.

With those caveats in place, the convergence of three different model specifications on a positive publication-history association is consistent with the systematic review of Leiviska et al. (23), which identified authentic research participation, most notably student placements within clinical research programs, as one of the most effective strategies for bridging the research–practice nexus. It also echoes the competency-based evidence reported by Grande et al. (16) and the meta-analytic findings of Jeong et al. (2), both of which positioned scholarly productivity as a proximal correlate of EBP competence. From a curricular standpoint, our data are consistent with the proposition that research participation acts as a boundary-crossing activity that engages information literacy, critical appraisal, and evidence application simultaneously, although a longitudinal design will be required to confirm that this engagement pattern reflects skill development rather than self-selection of more engaged learners into research opportunities.

### 5.4 Demographic correlates: sex, age, and academic performance

Male sex was independently associated with lower total EBP engagement in the adjusted model. While this finding contrasts with reports in which demographic factors exerted no significant influence (27), it is consistent with evidence that female nursing students in Gulf contexts engage more frequently with academic resources and collaborative learning environments (4, 5). Because sex distribution differs between the two strata of our sample, and because clinical-rotation assignments and peer-learning networks remain partly sex-segregated in the regional educational context, causal inference is inappropriate; the association should motivate targeted engagement strategies rather than deficit-based interpretations. The 22–25-year age band was likewise associated with higher scores, most plausibly because this stratum coincides with senior academic standing and internship placement, a positional rather than maturational association (3, 8). Finally, GPA showed no relationship with total engagement, in line with the contention of Purabdollah et al. (25) that conventional summative assessment indexes memorization and procedural recall more than the critical-thinking and inquiry skills central to EBP. This null finding has direct implications for curricular assessment design, developed in §6.2 below.

### 5.5 Limitations

Several limitations should be acknowledged when interpreting the findings of this study. First, cross-sectional design permits comparison between groups at a single point in time but does not allow conclusions about causality or developmental change. Longitudinal approaches that follow the same participants from the academic phase into clinical training would be required to determine whether the observed differences reflect true progression rather than cohort-related variation. Second, the complete overlap between internship status and prior research experience reflects a structural characteristic of the curriculum rather than a sampling imbalance. As a result, the primary regression model captures their combined influence on EBP engagement, while only supplementary analyses provide partial insight into their independent effects. Third, data collection relied on self-reported responses using the S-EBPQ (17). Although the instrument demonstrates strong psychometric properties, self-report measures may be influenced by social desirability bias and may not fully correspond to actual clinical performance. Objective assessments of evidence appraisal, literature-search competence, and clinical decision-making were not included and represent an important area for future work. Fourth, the relatively modest proportion of explained variance (*R*^2^ = 10.6%) suggests that relevant contextual and organizational factors were not captured. Variables such as mentorship quality, supervisory support, workplace culture, and access to clinical resources have been identified in prior research as important determinants of EBP engagement and should be incorporated in future multilevel studies (13, 14, 24).

Fifth, the study was conducted within a single institutional context Northern Border University, which may limit the generalizability of the findings to other educational or healthcare settings with different curricular structures, language contexts, or clinical training models. Broader, multi-site investigations are therefore recommended. Sixth, although the questionnaire was administered in English and underwent expert review and pilot testing, a formally translated and psychometrically validated Arabic version would further strengthen its applicability in similar contexts. Finally, the study did not examine associations between EBP engagement and patient-level outcomes, which remain a critical benchmark for evaluating the real-world impact of evidence-based practice education (23).

## 6 Conclusion

In this single-institution comparative cross-sectional study, nursing interns reported higher levels of self-reported EBP engagement than fourth-year students across the three behavioral domains of the S-EBPQ, while fourth-year students reported somewhat more favorable attitudes toward EBP. Prior research publication was an independent positive correlate of total engagement in the primary model and in two pre-specified sensitivity analyses, and male sex was independently associated with lower scores; GPA was unrelated to the outcome after adjustment. Because the design is cross-sectional and because intern status co-occurs perfectly with prior research participation in this curriculum, these findings should be read as stage-related differences and as associations rather than as evidence of developmental change or as isolated causal effects of internship or research participation. They are nevertheless consistent with, and, against the *a priori* interpretive framework adopted here, educationally meaningful in relation to a broader literature that frames EBP engagement as a contextually situated capability shaped by the interaction of curriculum design, clinical learning environments, and authentic scholarly engagement. Bridging the academic–clinical gap is therefore likely to require curricular continuity rather than curricular addition: embedding evidence-literacy and research-engagement opportunities longitudinally from the academic years into the internship year, and reinforcing them through mentorship, protected time, and access to point-of-care evidence resources. Longitudinal multi-site studies that incorporate objective assessment of EBP skills, organizational context measures, and downstream patient-outcome indicators are needed to confirm and extend the present associational findings.

## 7 Implications

### 7.1 Theoretical implications

The co-occurrence of higher behavioral EBP engagement and lower attitudinal scores among interns observed in this study is difficult to reconcile with unidirectional belief-then-behavior models of competency formation, and it is more readily accommodated by contextual, practice-based theorizations in which behavior and affect are mutually constituted within enabling or constraining clinical ecosystems (23, 24). Our findings additionally support a repositioning of research participation, and completed dissemination, as a formative pedagogical experience that integrates the five steps of EBP, rather than as a downstream output of scholarly activity. Incorporating research-engagement indicators into next-generation EBP-competency frameworks, alongside attitude and behavioral domains, may therefore be warranted. The framework, however, requires validation in longitudinal and multi-site designs before it can be advanced confidently.

### 7.2 Educational implications

Nursing faculties should restructure curricula to embed evidence literacy longitudinally rather than as an isolated research module (3, 6). Bhatarasakoon, et al., (7) offer a particularly apposite pedagogical framework in this regard, arguing that effective undergraduate EBP teaching must move beyond procedural instruction to cultivate information literacy, critical inquiry, and reflective reasoning as integrated knowledge needs, and that this is achieved through scaffolded, inquiry-driven teaching strategies that explicitly link classroom learning to clinical decision-making. Practical curricular recommendations, drawing on that framework and on the present findings, include four interlocking elements. First, case-based and problem-based learning should be introduced in early academic semesters to cultivate inquiry habits before the clinical phase begins (33). Second, mentoring student research with explicit dissemination expectations, whether through peer-reviewed publication, conference presentation, or institutionally recognized scholarly output as defined in §2.3.5, should be embedded into the curriculum rather than left to elective initiative (16). Third, scaffolded, multimodal teaching strategies, including structured journal clubs, reflective EBP portfolios, and supervised search-and-appraisal exercises, should be deployed across the academic years and into internship, with explicit attention to learners for whom English is a second academic language (29). Fourth, assessment blueprints should be redesigned so that critical appraisal, evidence retrieval, and applied evidence use are weighted alongside knowledge recall, addressing the disconnect between GPA and EBP engagement (25). Taken together, these recommendations align with the theory-to-practice bridging strategy articulated by Bhatarasakoon et al. (7), and they reframe EBP education as a continuous developmental thread rather than a discrete curricular event.

### 7.3 Practice and policy implications

Healthcare organizations and, at the national level, the Saudi Commission for Health Specialties should institutionalize the structural enablers that the present findings, together with the broader literature, implicate as conditions for sustained EBP engagement: protected time for evidence retrieval during clinical shifts, reliable point-of-care database access, nurse-led EBP committees with explicit mandates, and preceptor-development programs that prepare clinical educators to model transparent evidence-based reasoning in front of trainees (31, 32). Alignment between national policy frameworks such as the quality improvement priorities outlined in Saudi Vision 2030 and undergraduate educational outcomes is essential for maximizing the impact of nursing education. Establishing formal connections between university-level evidence-based practice (EBP) competencies and ministry-level workforce quality indicators could enhance the return on educational investment. Such integration would also foster an enabling institutional environment that supports the sustainability of curricular reforms, ensuring that competency development extends beyond the academic setting and is continuously reinforced within clinical practice.

## Data Availability

The datasets presented in this article are not readily available because the dataset is provided as supplementary material in anonymized form. All direct identifiers were removed prior to sharing, and the data are restricted to variables approved under the study’s ethical clearance. Redistribution or use of the data for purposes beyond scientific research should occur in accordance with institutional ethical guidelines and applicable regulations. Requests to access the datasets should be directed to Fathia.hassan@nbu.edu.sa.
